# Breast mucoepidermoid carcinoma: about a case report

**DOI:** 10.1016/j.ijscr.2025.111402

**Published:** 2025-05-07

**Authors:** Imane Tazi, Soumaya Ech-charif, Ismail Boujida, Mouna Khmou, Youssef Mahdi, Basma El Khannoussi

**Affiliations:** Department of Pathology of the National Institute of Oncology, Ibn Sina University Hospital Center, Rabat 10100, Morocco; Faculty of Medicine and Pharmacy of Rabat, Mohamed V University, Morocco

**Keywords:** Mucoepidermoid carcinoma, Triple negative, Breast cancer

## Abstract

**Introduction and importance:**

Breast mucoepidermoid carcinoma (MEC) is a very rare entity and usually underdiagnosed by the pathologists.

**Case presentation:**

54-year-old woman with a single palpable mass on the left breast with no nipple discharge. She underwent tumoretomy. Microscopic examination revealed a well circumstanced intracystic proliferation consisted of mucoid, epidermoid and intermediate cells. The tumor cells were positive for ER, PR but negative for HER2.

**Clinical discussion:**

Salivary glands like neoplasms are described in the breast, and only represent 0.2 % to 0.3 % of breast carcinomas. They usually fall under the triple negative breast carcinomas. However some cases were described in the literature with a luminal A profile. The grading score used in the salivary glands based on the presence or lack thereof nuclear anaplasia, necrosis, high mitotic activity and perineural or lymphovascular invasion seems more appropriate in this rare entity.

**Conclusions:**

It is crucial for pathologists to think of this type of Breast carcinomas, especially when there is mucoid, epidermoid and intermediate cells and it does not have to fall under triple negative breast carcinomas.

## Highlights

• Mucoepidermoid carcinoma (MEC) is a very rare entity in the breast.
• Breast mucoepidermoid carcinoma (MEC) is not always included among triple negative breast cancers (TNBC).
• The score used in the salivary glands seems more appropriate in this entity.

## 1 Introduction

Breast cancer is a frequent aggressive tumor with 23.8 % of all cancers diagnosed in women worldwide in 2022, and approximately 38.8 % in Morocco [1]. The muco epidermoid carcinoma (MEC) is a rare subtype with a frequency of 0.2 % to 0.3 % [2].

Tumors of the mammary gland and the salivary gland share the same features due to their common ectodermal origin [3].

It is typically presented with a basal phenotype: negative for estrogen receptor (ER), progesterone receptor (PR), and human epidermal growth factor receptor 2 (HER-2) [4]. However recently there have been some cases with positive hormonal receptors.

We present a case of breast MEC that is positive for ER and PR, and review the literature focused on pathological findings, and especially highlight the pathological pitfalls. This case was presented in accordance with the updated consensus Surgical Case Report (SCARE) guidelines [5].

## 2 Case report

A 54 years old woman, with no prior otolaryngologic history, was referred to our institution for discovering a palpable mass on the left breast.

Physical examination showed a 3-cm lump at the junction of the outer quadrants of the left breast, no skin changes or nipple discharge were observed. The axillary lymph nodes were not palpable.

**Fig. 1:**
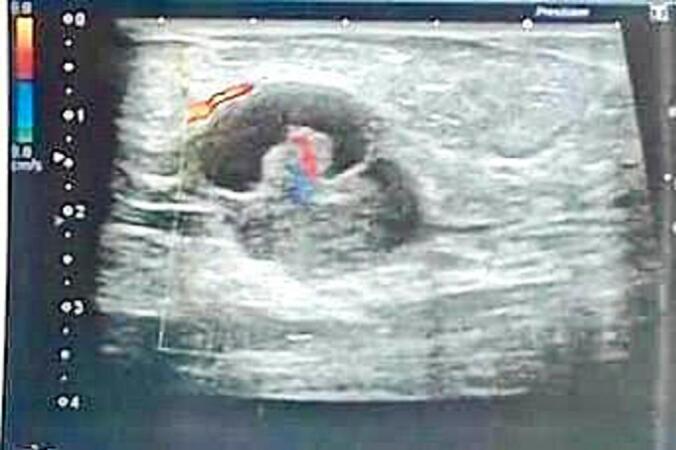
Ultrasonography imaging Cystic dilatation of an intraductal canal centred by a tissue materiel measuring 26.5mmx13.6 mm, categorized according to BIRADS system (Breast Imaging Reporting and Data System (BI-RADS) category 4c).

Mammography and ultrasonography showed a cystic dilatation of an intraductal canal of the left lower outer quadrant centred by a tissue materiel measuring 26.5mmx13.6 mm, categorized according to BIRADS system (Breast Imaging Reporting and Data System (BI-RADS) category 4c (Fig. 1).

Ultrasound-guided core-needle biopsy was performed and revealed at first a morphological appearance of mastitis. The pet scan showed a hypermetabolic left breast nodule, at the lower outer quadrant. No other lesions were found.

A tumorectomy was realised and showed a white well limited nodule, measuring 25 × 17 mm.

**Fig. 2:**
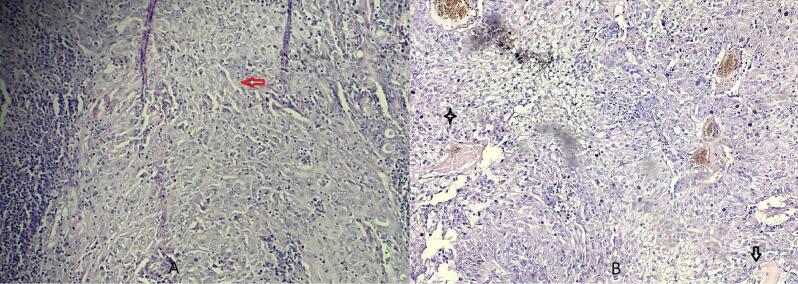
Hematoxylin eosin stain (Ax200 Bx400) Well circumscribed intracystic proliferation arranged in solid strip, consisted of mucoid cells that were large and round with vacuolar pale cytoplasm (black arrow). The other cells were polygonal with eosinophilic cytoplasm, and round small nuclei (red arrow = intermediate cells, star = epidermoid). (For interpretation of the references to colour in this figure legend, the reader is referred to the web version of this article.)

Histopathological examination with hematoxylin eosin stain showed a well circumscribed intracystic proliferation arranged in solid strip, consisted of mucoid cells that were large and oval with vacuolar pale cytoplasm. The other cells were polygonal with eosinophilic cytoplasm, and round small nuclei. No necrosis, lymphovascular and nerve invasion were observed (Fig. 2).

**Fig. 3:**
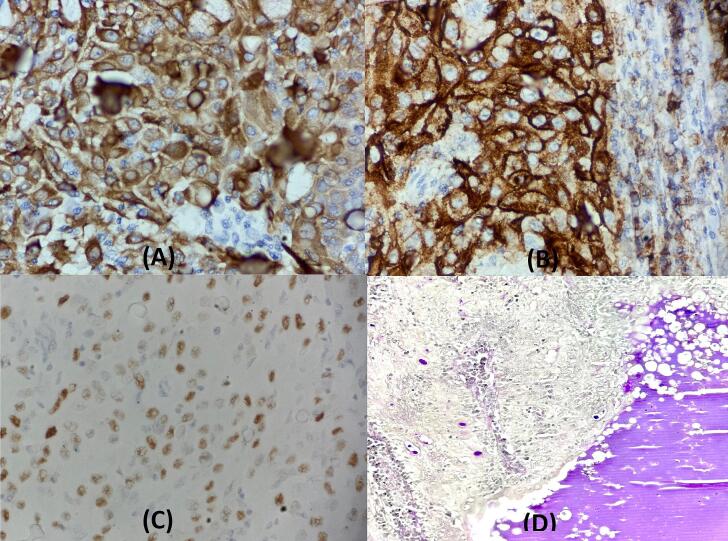
A = CK7x400,B = MUC1X400, C=P63x400, D = PAS x200 The mucoid cells expressing positive stain for CK7 (A), MUC1(B) and PAS (D) and negative for P63. The outer cells are polygonal and eosinophilic epidermoid cells are positive for P63. (C).

PAS stain showed an important mucosecretion. The tumor cells were positive for CK7, MUC1 and P63 (Fig. 3).

**Fig. 4:**
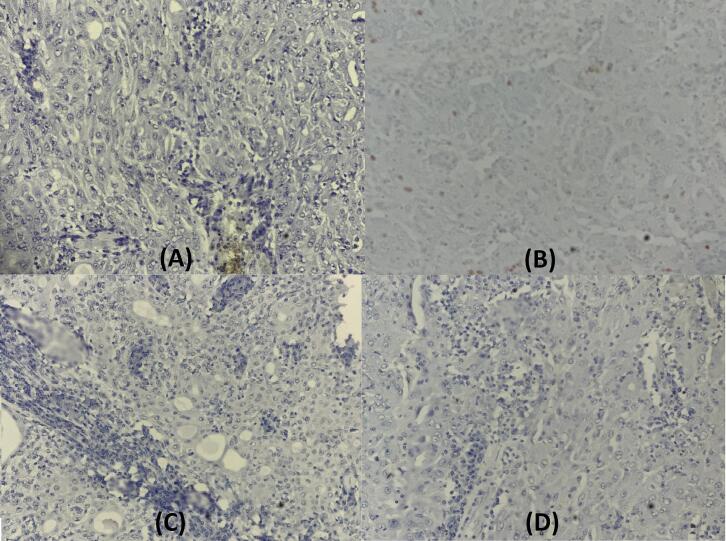
Stain for A = ER x200, B = PRx200, C=HER2 x 200, D = ki67 x200 The tumor cells showing weak nuclear stain for ER, and PR (A and B). And no stain for HER2(C). The index proliferation ki67 is very low (D).

Less than 10 % of the tumor cells showed a low staining for ER,30 % expressed PR and no staining for HER2.The proliferation index (ki67) was very low (Fig. 4).

After the multidisciplinary meeting, the medical team decided to proceed with a lymph node dissection. The microscopic examination showed no lymph node metastasis.

The multidisciplinary consultation committee recommended initiating chemotherapy associated with radiotherapy and hormonotherapy for the patient and established a clinical follow-up plan. To date, the patient has not shown any signs of recurrence after 8 months of monitoring.

## 3 Discussion

MEC is one of the most aggressive tumors in salivary glands, but it can also occur in other organs such as the breast, thyroid, esophagus, bronchi, pleura, forearm, tonsils, colon, and thymus, with only a few cases reported [6].

Most reported breast MECs have been included among the triple-negative breast carcinomas, with only few cases being reported for the last 20 years (Table 1).

**Table 1:** Summary of published cases of breast MEC for the past 20 years.

Year	Country	Age	Tumor size and grade	IHC profile	Treatment	Outcome	Sources
2006	Japan	54	2,5 cm, Low	Triple negative	Mastectomy + ALND	NED at 36 months	Horii R, Akiyama F, Iwaya K, et al. Mucoepidermoid carcinoma of the breast: a case report with a review of the literature. Pathol Int. 2006;56(6):333–337
2006	Spain	69	7,5 cm, High	Triple negative	MRM + ALND	NED at 54 months	Gómez-Aracil V, Mayayo E, Azua J, Aranda FI. Mucoepidermoid carcinoma of the breast: an unusual triple-negative tumor. Virchows Arch. 2006;449(4):460–463
2007	Czech Republic	63	1,8 cm, High	Triple negative	Simple mastectomy + ALND + chemoRT	NED at 18 months	Hornychová H, Ryska A, Cermáková E. Mucoepidermoid carcinoma of the breast: two case reports and review of the literature. Cesk Patol. 2007;43(4):181–185
2007	Czech Republic	30	8 cm, Low	Triple negative	MRM + chemoRT	NED at 60 months	Hornychová H, Ryska A, Cermáková E. Mucoepidermoid carcinoma of the breast: two case reports and review of the literature. Cesk Patol. 2007;43(4):181–185
2009	USA	49	4 cm, Intermediate	Triple negative	MRM + ALND + chemotherapy	NED at 8 months	Camelo-Piragua S, Argani P, Otis CN, et al. Primary mucoepidermoid carcinoma of the breast: a case report and review. Int J Surg Pathol. 2009;17(4):310–315
2011	Turkey	69	10 cm, High	Triple negative	MRM + ALND + chemoRT	NED at 12 months	Basbug M, Uyaroglu MA, Sari I, et al. Primary high-grade mucoepidermoid carcinoma of the breast: report of a case. Diagn Pathol. 2011;6:126
2013	Turkey	40	5.5 cm, High	Triple negative	MRM + ALND + chemotherapy	NED at 5 months	Türk Ö, Yıldırım Y, Güler A, et al. High-grade mucoepidermoid carcinoma of the breast: a rare triple-negative tumor. Turk Patoloji Derg. 2013;29(3):210–213
2013	Poland*	80	4 cm, High	Triple negative	Lumpectomy	NED (time NR)	Palermo R, García-Prats MD, et al. Mucoepidermoid carcinoma of the breast in an elderly woman: a rare case. Pol J Pathol. 2013;64(4):287–290
2016	India	34	4-5 cm, Low	Triple negative	MRM + ALND + chemoRT	NED at 24 months	Arun Kumar T, Manjunath GV, Shankar K, et al. Low-grade mucoepidermoid carcinoma of breast: an unusual triple-negative case. Indian J Pathol Microbiol. 2016;59(4):531–533
2016	Japan	71	2 cm, Intermediate	Triple negative	Mastectomy + SLNB	NED (time NR)	Fujino T, Mori T, et al. Mucoepidermoid carcinoma of the breast with basal-like features: a case report. J Med Case Rep. 2016;10:275
2017	Mexico	86	6 cm, Low	Triple negative	MRM	NED at 3 months	Sherwell-Cabello C, Flores RM, et al. Mucoepidermoid carcinoma of the breast presenting as a breast abscess. Breast J. 2017;23(3):345–346
2017	China	39	1.5 cm, Low	Triple negative	MRM + ALND	NED at 156 months	Cheng Z, Wang L, Zhang Q. Low-grade mucoepidermoid carcinoma of the breast: a case series with follow-up and review. Diagn Pathol. 2017;12:69
2017	China	49	1.5 cm, Low	Triple negative	MRM + ALND	NED at 41 months	Cheng Z, Wang L, Zhang Q. Low-grade mucoepidermoid carcinoma of the breast: a case series with follow-up and review. Diagn Pathol. 2017;12:69
2017	China	66	1.3 cm, Low	Triple negative	Simple mastectomy + SLNB	NED at 9 months	Cheng Z, Wang L, Zhang Q. Low-grade mucoepidermoid carcinoma of the breast: a case series with follow-up and review. Diagn Pathol. 2017;12:69
2017	China	61	3 cm, Low	Triple negative	Simple mastectomy + SLNB	NED at 4 months	Cheng Z, Wang L, Zhang Q. Low-grade mucoepidermoid carcinoma of the breast: a case series with follow-up and review. Diagn Pathol. 2017;12:69
2018	Jordan	73	NA, Low	Triple negative	Lumpectomy + ALND	NED (time NR)	Burghel GJ, Abushalha M, et al. Mucoepidermoid carcinoma of the breast with extensive mucinous component: a rare variant. Diagn Cytopathol. 2018;46(8):707–710
2019	China	60	1.9 cm, Low	Triple negative,MAML2+	Lumpectomy	NED at 60 months	Yan X, Li Y, Liu Y, et al. Mucoepidermoid carcinoma of the breast: clinicopathological analysis of a rare subtype. Ann Diagn Pathol. 2019;40:114–117
2020	China	42	2.6 cm, Low	Triple negative	MRM	NED at 12 months	Ye Q, Zhang S, et al. Breast mucoepidermoid carcinoma: case report and literature review. Medicine (Baltimore). 2020;99(31):e21347
2022	China	38	NA, Low	Triple negative	Lumpectomy	NED at 6 months	Chen J, Li D, Wang H, et al. Primary mucoepidermoid carcinoma of the breast: report of a rare case. Int J Clin Exp Pathol. 2022;15(4):395–400
2022	USA	50	NA, Low	ER+, PR-, HER2- (focal luminal differentiation)	Lumpectomy + SLNB + RT + hormonotherapy	NED	Bui MM, Bose S. Low-grade mucoepidermoid carcinoma arising in breast adenomyoepithelioma: a case report. Am J Surg Pathol. 2022;46(5):653–657
2022	S. Korea	47	3.2 cm, Intermediate	ER+, PR-, HER2-, Ki high	Lumpectomy + SLNB + chemo + RT + tamoxifen	NED at 37 months	Bak M, Kim SH, et al. Intermediate-grade mucoepidermoid carcinoma of the breast in a young patient: case report. J Breast Cancer. 2022;25(1):79–83
2023	India	73	“Absess like”, High	Triple negative	MRM + ALND	NED at 3 months	Gupta S, Yadav A, Goyal P, et al. Mucoepidermoid carcinoma of the breast mimicking an abscess: a rare entity. Indian J Surg Oncol. 2023;14(1):183–186
2023	Belgium	58	2 cm, High	ER (weak+5–10 %) HER2 + (3+ amplified)	Simple mastectomy + HER2 therapy	NED	Floris G, Christiaens MR, et al. Mucoepidermoid carcinoma of the breast with HER2 positivity: a rare case with literature review. Histopathology. 2023;82(3):534–540
2024	USA	67	3.1 cm, Intermediate	ER+, PR-, HER2-	Lumpectomy + SLNB + RT + hormonotherapy	NED at 6 months	Zhang Y, Sun W, et al. Low-grade mucoepidermoid carcinoma of the breast with endocrine features: report of a case. Diagn Pathol. 2024;19(1):21
2024	China	47	5 cm, High	Triple negative, MAML2 -	MRM + ALND	NED at 12 months	Zhang H, Liu Y, Wu Q, et al. High-grade mucoepidermoid carcinoma of the breast without MAML2 fusion: a case report. Medicine (Baltimore). 2024;103(12):e34567

ALND: axillary lymph node dissection MRM: modified radical mastectomy chemoRT: chemoradiotherapy SLNB: sentinel lymph node biopsy NED: No evidence of disease.

Recently ER, PR positivity was described in a subpopulation of the neoplastic cells of low-and intermediate-grade breast MEC [7]. Our case also expressed ER and PR.

The clinical features include female patients aged 29 to 80 years [8]. It typically presents as unifocal breast nodules or cystic lesions, ranging in size from 6 mm to 11 mm [8]. MEC can also present as a nodule or with nipple discharge when located in the retro-areolar region [9].

Salivary gland-like tumors of the breast encompass a broad range of entities. They can be categorized as follows: 1) tumors exhibiting pure myoepithelial cell differentiation, such as pure benign and malignant myoepitheliomas; 2) tumors with mixed epithelial and myoepithelial cell differentiation, including pleomorphic adenoma, adenomyoepithelioma, and adenoid cystic carcinoma; and 3) tumors with pure epithelial cell differentiation, such as acinic cell carcinoma, oncocytic carcinoma, mucoepidermoid carcinoma, and polymorphous adenocarcinoma [10].

These cells grow solid, cystic or mixed, occasionally papillary or adenoid [6].

Breast MECs is composed of three cell types: Mucoid cells have abundant cytoplasm with a nuclei positioned at the periphery of the cell. Intermediate cells are larger with an eosinophilic cytoplasm. The epidermoid cells are angulated and also exhibit eosinophilic cytoplasm but do not undergo true keratinization [11].

It is more appropriate to use the grading system in salivary glands than The Elston and Ellis. It defines high-grade MEC by the presence of at least one of the following features: nuclear anaplasia, necrosis, high mitotic activity, and perineural or lymphovascular invasion. These criteria were utilized by Auclair et al., who also considered the percentage of the intracystic component to classify MEC of the oral cavity into three grades (low, intermediate, and high), which are correlated with prognosis [12].

Most mucoid cells express low molecular weight keratins such as CK7, while epidermoid or polygonal cells are positive for high molecular weight keratins like CK5/6 [13].

Palermo et al. found that MUC6 and MUC5AC stained the invasive component, while MUC1 and MUC6 were present in the in situ component [14]. The basaloid, intermediate, and epidermoid cells are stained with CK14, p63, and high molecular weight CKs (100−102), while mucous cells are stained with CK7 [10].

Low-grade tumors usually grow cystic, with well circumscribed and rich with mucinous cells, whereas high-grade tumors are more solid, less well-defined, and contain more intermediate and epidermoid cells [15].

From a molecular perspective, a rearrangement of the MAML2 gene is commonly described in MECs of salivary glands [3], though we did not perform molecular analysis.

Differential diagnosis includes a salivary gland tumor metastasis in the breast, which is rare. Invasive lobular carcinoma is excluded on the basis of the focal and weak positivity for CK7, strong positivity for BCL-2 and negativity for ER and PR [10].

Sherwell-Cabello et al. reported that patients with low levels of hormone receptor expression had a good prognosis [16]. This suggests that the disease may be hormone-dependent, making endocrine therapy a potential treatment option. Nakano et al. found that patients with MAML2 gene rearrangement had a better prognosis [17].

The treatment procedures of MECs of the breast are similar to any invasive breast cancer, including surgery, chemotherapy, radiotherapy, targeted therapies, immunotherapy and hormonal therapy. It depends on several factors, notably the patient's clinical condition, the grade and size of the tumor and the presence or not of lymph node metastasis [2].

## 4 Conclusion

Triple negative breast carcinomas are usually associated with bad prognosis and severe clinical presentation. Breast MEC is a very rare entity that is usually under diagnosed, and as we presented in our case, cannot always be triple negative. Every pathologist should recall it especially when mucoid cells are present. This entity is distinguished from other triple negative breast cancer by its good prognosis.

## CRediT authorship contribution statement

Imane tazi and Soumaya Ech-charif were responsible for the patient's diagnosis and clinical management, and wrote the manuscript. Ismail Boujida, Mouna Khmou, Youssef Mahdi, Basma El Khannoussi contributed to the analysis, supervision, writing, reviewing, and editing of the manuscript for intellectual content. All authors have read and approved the final manuscript

## Consent

Informed verbal consent was obtained from the patient.

## Ethical approval

It is not necessary to obtain specific ethical approval from the National institute of oncology Hospital for this situation because the case report does not contain any personal information.

The national institute of oncology hospital does not require ethical approval because we do not provide personal information.

## Guarantor

Is the corresponding author: Imane Tazi.

## Research registration number

Not applicable.

## Funding

No funding source was needed.

## Declaration of competing interest

The authors declare that they have no competing interests.
